# Inhibitory effects of fucoidan on NMDA receptors and l-type Ca^2+^ channels regulating the Ca^2+^ responses in rat neurons

**DOI:** 10.1080/13880209.2018.1548626

**Published:** 2019-02-08

**Authors:** Hong Wu, Shuibo Gao, Susumu Terakawa

**Affiliations:** a Laboratory of Cell Imaging, Henan University of Chinese Medicine, Zhengzhou, China;; b Photon Medical Research Center, Hamamatsu University School of Medicine, Hamamatsu, Japan

**Keywords:** Cortical and hippocampal neuron, Ca^2+^ transient, sulphated polysaccharide, mRNA expression of PR1/PR2

## Abstract

**Context:** Fucoidan, a sulphated polysaccharide extracted from brown algae [*Fucus vesiculosus* Linn. (Fucaceae)], has multiple biological activities.

**Objective:** The effects of fucoidan on Ca^2+^ responses of rat neurons and its probable mechanisms with focus on glutamate receptors were examined.

**Materials and methods:** The neurons isolated from the cortex and hippocampi of Wistar rats in postnatal day 1 were employed. The intracellular Ca^2+^ responses triggered by various stimuli were measured *in vitro* by Fura-2/AM. Fucoidan at 0.5 mg/mL or 1.5 mg/mL was applied for 3 min to determine its effects on Ca^2+^ responses. RT-PCR was used to determine the mRNA expression of neuron receptors treated with fucoidan at 0.5 mg/mL for 3 h.

**Results:** The Ca^2+^ responses induced by NMDA were 100% suppressed by fucoidan, and those induced by Bay K8644 90% in the cortical neurons. However, fucoidan has no significant effect on the Ca^2+^ responses of cortical neurons induced by AMPA or quisqualate. Meanwhile, the Ca^2+^ responses of hippocampal neurons induced by glutamate, ACPD or adrenaline, showed only a slight decrease following fucoidan treatment. RT-PCR assays of cortical and hippocampal neurons showed that fucoidan treatment significantly decreased the mRNA expression of NMDA-NR1 receptor and the primer pair for l-type Ca^2+^ channels, PR1/PR2.

**Discussion and conclusions:** Our data indicate that fucoidan suppresses the intracellular Ca^2+^ responses by selectively inhibiting NMDA receptors in cortical neurons and l-type Ca^2+^ channels in hippocampal neurons. A wide spectrum of fucoidan binding to cell membrane may be useful for designing a general purpose drug in future.

## Introduction

Fucoidan is a group of sulphated fucose-containing polysaccharides obtained from brown algae. Fucoidan has multiple biological activities, including anticancer, immune and clotting modulation, anti-inflammation, etc. (Abudabbus et al. 2017; Li et al. 2017; Takahashi et al. 2018). Additionally, the neuroprotective effects of fucoidan had been confirmed both *in vivo* and *in vitro*. Fucoidan could protect rat cholinergic basal forebrain neurons against β-amyloid-induced death *in vitro* (Jhamandas et al. 2005). Luo et al. (2009) demonstrated that fucoidan significantly reduced dopaminergic neuron death induced by 1-methyl-4-phenylpyridinium (MPP(+)) through inhibiting lipid peroxidation and reduction of antioxidant enzyme activity. Moreover, researchers also showed that fucoidan effectively improved the behavioural deficits of animal models with dopaminergic neuronal damage (Luo et al. 2009; Cui et al. 2012; Zhang et al. 2014). Importantly, under conditions of disease and injury, excessive buildup of intracellular Ca^2+^ could induce neuronal damage and death involving central nervous system (CNS) disorders (Fujikawa 2015). However, the effects of fucoidan on influx of Ca^2+^ ions in neurons remain unclear.

Our preceding study showed that fucoidan had inhibitory effects on the activities of G-protein-coupled receptors regulating the Ca^2+^ responses (Wu et al. 2018). In that study, we mainly used HeLa cells to observe the Ca^2+^ responses induced by various agonists such as histamine, adenosine-5′-triphosphate (ATP), compound 48/80 and acetylcholine. Histamine and compound 48/80 are the agonists only for stimulating G-protein-coupled receptors to induce the Ca^2+^ responses (Higashijima et al. 1988; Hill et al. 1997). Acetylcholine induces the Ca^2+^ responses via both ionotropic and metabotropic receptors (Zuccolo et al. 2017; Ipsen et al. 2018). We also confirmed that there was no ionotropic cholinergic receptor expressed in HeLa cells. Another agonist used was ATP which belongs to purinergic receptor agonist (Khakh et al. 2001; Abbracchio et al. 2006). Our data showed that ionotropic purinergic receptors were insensitive to fucoidan. We, therefore, tentatively concluded that the effects of fucoidan on the Ca^2+^ responses were very consistent with each other in a sense that fucoidan had inhibitory effects on the three types of metabotropic receptors in cultured HeLa cells. Noteworthily, purinergic ionotropic receptors were expressed in the cell membrane of HeLa cells, but they were insensitive to function, leading to the notion that fucoidan suppresses metabotropic receptors but not the ionotropic receptor. Neurons which express glutamate receptors (GluRs) including both ionotropic type (iGluRs) and metabotropic type (mGluRs) (Traynelis et al. 2010; Julio-Pieper et al. 2011), are the good candidates for extending our comparative studies on effects of fucoidan on various membrane receptors.

In the present study, we investigated the effects of fucoidan mainly on glutamate receptors in cortical and hippocampal neurons. In an attempt to induce the Ca^2+^ responses, iGluRs agonists such as *N*-methyl-d-aspartate (NMDA) and α-amino-3-hydroxy-5-methyl-4-isoxazolepropionic acid (AMPA) in the presence of extracellular Ca^2+^ and mGluRs agonists such as quisqualate and (1S,3R)-1-aminocyclopentane-1,3-dicarboxylic acid (ACPD) in the absence of extracellular Ca^2+^ were used. Glutamate, an agonist for both iGluRs and mGluRs, was also used in the presence or absence of extracellular Ca^2+^. In contrast to our expectation, fucoidan showed a very selective inhibition of only the NMDA receptor (NMDAR) (one of iGluRs) but not other types of receptors examined.

## Materials and methods

### Materials

Fura-2 acetoxymethyl ester (Fura-2/AM) was purchased from Dojindo Laboratories (Kumamoto, Japan). Horse serum was from Life Technology (Grand Island, NY). B27 supplement was purchased from Invitrogen (Grand Island, NY). Quisqualic acid (quisqualate) was from Wako Pure Chemical Industries, Ltd. (Osaka, Japan). Fucoidan (purified from *Fucus vesiculosus* Linn. (Fucaceae)), poly-d-lysine, cytosine β-d-arabinofuranoside (cytosine arabinoside), ACPD, Bay K8644, AMPA and NMDA were obtained from Sigma-Aldrich (St. Louis, MO).

### Primary cultures of the cortical neurons and hippocampal neurons

The animal procedures were approved by the institutional review committee and were kept in accordance with the Guide for the Care and Use of Laboratory Animals at Hamamatsu University School of Medicine. Wistar rats in postnatal day 1 purchased from a local animal centre (Japan SLC Inc., Shizuoka, Japan) were anaesthetized with ether, and then the cortex or hippocampus was dissected from their brain. The tissue was cut into small pieces with a pair of scissors, and then the pieces were dispersed into a cell suspension in the neuron medium using plastic pipette. The density and viability of the isolated cells was determined using a hematocytometer and trypan blue staining, respectively. These cells were seeded into 35-mm poly-d-lysine coated glass-bottomed dishes at a density of about 2 × 10^5^ cells per dish. The cell suspension (around 200 µL) in the dishes was incubated in a humidified incubator with 5% CO_2_ for 20 min at 37 °C to allow cell adhesion, and then, additional neuron medium (2 mL) was supplemented into the dishes. On day 1, 2 and 6, 500 µL of medium was replaced with 700 µL of fresh neuron medium. The neuron medium was a mixture of Neurobasal A (GIBCO No. 10888, Invitrogen, Tokyo, Japan) containing: 10% horse serum; 2 mM l-glutamine (GIBCO No. 25030-149, Invitrogen, Tokyo, Japan); 10 µg/mL gentamycin (G-1264, Sigma, Darmstadt, Germany); and 2% B27 supplement. From day 2, 1 µM of cytosine arabinoside was added into neuron medium. The cells cultured for 10–14 days were used for following experiments.

### Measurement of intracellular Ca^2+^

The intracellular calcium concentration ([Ca^2+^]*_i_*) was measured using procedures similar to those we used for HeLa cells (Wu et al. 2018). In brief, after 10–14 days of culture, cortical and hippocampal neurons were incubated with fluorescent Ca^2+^ indicator dye Fura-2/AM (2.5 µM) for 20 min at 37 °C in the dark and then rinsed twice with artificial cerebro-spinal fluid (aCSF) for calcium imaging. The aCSF contained (in mM): NaCl, 140; KCl, 5; CaCl_2_, 2; MgCl_2_, 1.2; glucose, 10; and HEPES (pH 7.2) 10. Fura-2/AM loaded cells were placed on the stage of an inverted microscope (IX 70; Olympus, Tokyo, Japan) and were illuminated at wavelengths of 340 and 380 nm every 3 s alternately. An intensified charge-coupled device (CCD) camera (C4742-95, Hamamatsu Photonics, Hamamatsu, Japan) was used to capture the calcium images at 510 nm with a 40× objective lens (UApo 40×/340, NA 0.9, Olympus, Tokyo, Japan). In the control, the agonists, such as glutamine, NMDA, and so on, were applied for 3 min after recording the baseline for 3 min. In the test, fucoidan was added into the aCSF during the same baseline period (3 min), and then without removal of fucoidan, the agonists were further added into aCSF. The changes in [Ca^2+^]*_i_* in individual cells were determined by the 340/380 nm fluorescence ratio with an image-analysis software (Aquacosmos, Hamamatsu Photonics, Hamamatsu City, Japan). A calcium-free aCSF was also used, in which case, CaCl_2_ was removed and ethyleneglycol tetraacetic acid (EGTA) was added at 1 mM (pH 7.4).

### Real-time PCR

To identify the effects of fucoidan on the expressions of GluRs, real-time PCR (RT-PCR) was used to measure the mRNA expressions of iGluRs and mGluRs. Cortical and hippocampal neurons were serum-starved for 24 h and treated with 0.5 mg/mL fucoidan for 3 h. Total RNA was resolved in 10 μL of diethylpyrocarbonate-treated water, and 1 μg of each RNA samples was reverse-transcribed into cDNA by RT Easy^TM^ II Kit (first-strand cDNA for real-time PCR) (FOREGENE, Chengdu, China). RT-PCR was performed using Real Time PCR Easy^TM^-SYBR Green I Kit (FOREGENE, Chengdu, China) according to the manufacturer's instructions. The conditions consisted of an initial denaturation step at 95 °C for 2 min, followed by 40 cycles of 30 s denaturation steps at 95 °C, 30 s annealing step at 53.5 °C to 56.5 °C, and 1 min extension step at 72 °C. A final extension step of 5 min at 72 °C was also performed. PCR amplification products were analysed by electrophoresis on a 1% agarose gel. All PCR results were derived with cycle number producing a signal in the linear portion of the amplification curve. The primers were listed as follows (Table 1). The β-actin gene was used as the internal standard, and data were expressed as the ratio of iGluRs mRNA to β-actin mRNA or mGluRs mRNA to β-actin mRNA.

**Table 1.** PCR primer sequence.

Gene	Primer sequence
NR1	Fwd: 5′-GTTCGCCAACTACAGCATCATG-3′
	Rev: 5′-GACGTGGGTGCCATTGTAGAT-3′
GluR2	Fwd: 5′-CTTGAAGGCAATGAGCGCTAT-3′
	Rev: 5′-ACCCACAATGTTTGGCGATT-3′
GluR6	Fwd: 5′-GGTGTCTGTGGCCGTTCAA-3′
	Rev: 5′-GAAGCGCCAGGGTTTATGTC-3′
PR1/PR2	Fwd: 5′-CGAAGCTTCTTCATGATGAACATCTT-3′
	Rev: 5′-GCGGATCCATGTAGAAGCTGATGAA-3′
mGluR1	Fwd: 5′-CATCCCACAGATCGCCTATT-3′
	Rev: 5′-TGCCTGCAAAGTGTCAGAAG-3′
mGluR5	Fwd: 5′-CACTCTTGCCCAACATCAC-3′
	Rev: 5′-CACAGCGTACCAAACCTTC-3′
β-Actin	Fwd: 5′-AGCCATGTACGTAGCCATCC-3′
	Rev: 5′-ACCCTCATAGATGGGCACAG-3′

### Statistical analysis of the Ca^2+^ responses

For the quantification of Ca^2+^ responses, 10–20 cells were analysed in each observation field. All observations were repeated using 3–5 culture dishes. For statistical analyses, software implemented in Microsoft Excel 2010 was used. The integrated amplitude of the Ca^2+^ response was calculated by using an extended baseline for subtraction. The results were expressed as mean ± SEM.

## Results

### Effects of fucoidan on the Ca^2+^ responses induced by glutamate receptor agonists

**Figure 1.**
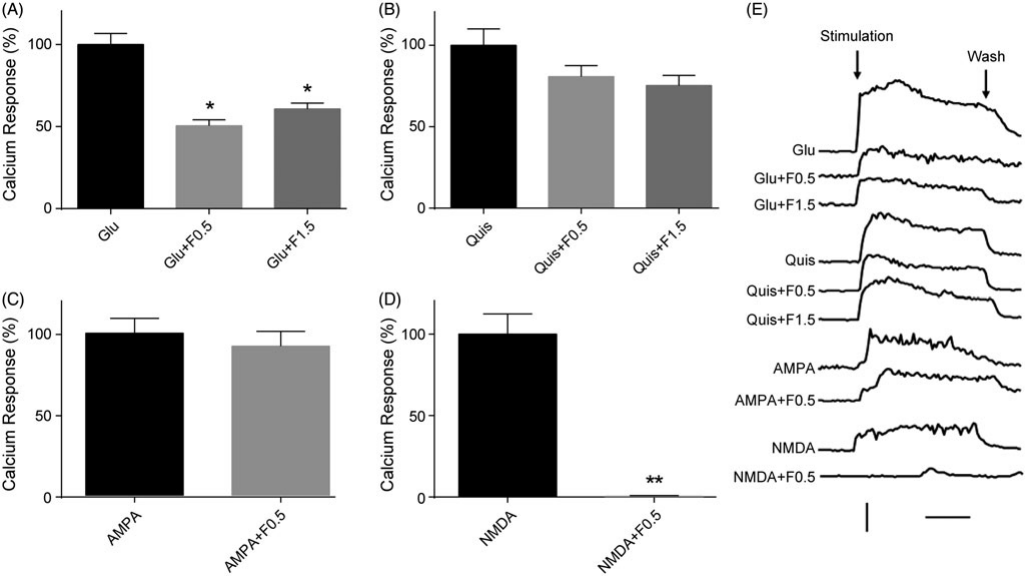
Effects of fucoidan on the Ca^2+^ responses induced by glutamate receptor agonists in the presence of extracellular Ca^2+^ in cortical neurons. (A–D) Quantification of the areas of Ca^2+^ response curves was taken from all records in each test. The ordinate is expressed in reference to the control value. Note that the column for quantification of the areas of Ca^2+^ response curves was taken from all records in each test. The ordinate is expressed in reference to the control value. Note that the column for NMDA application in the presence of fucoidan indicates zero level. The error bars represent the SEM. *p < 0.05 or **p < 0.01 vs. the untreated group. (E) Representative time course curves of the Ca^2+^ responses induced by diverse glutamate receptor agonists with or without fucoidan. In the control, the agonists were applied (for 3 min) after recording the baseline for 3 min. In the test, fucoidan was added to the aCSF during the same baseline period (3 min), and then without removal of fucoidan, the agonists were further added to aCSF. Glu: glutamate; Quis: quisqualate; F: fucoidan. Vertical bar, ratio of *F*_340_/*F*_380_ to be 1; horizontal bar, 1 min. *n* = 3.

Glutamate is the major excitatory neurotransmitter in CNS, acting through both ligands gated ion channels (iGluRs) and G-protein-coupled receptors (mGluRs) (Traynelis et al. 2010; Julio-Pieper et al. 2011). The ratio of iGluRs to mGluRs is variable due to different neuron types. Using primary cultured cortical and hippocampal neurons, the Ca^2+^ responses were examined by time-lapse imaging with Fura-2/AM (2.5 µM). The cells were preincubated with 0.5 and 1.5 mg/mL fucoidan and then stimulated with glutamate (50 µM). In the presence of extracellular Ca^2+^, glutamate exposure largely increased the [Ca^2+^]*_i_* in cortical neurons (Figure 1(A,E)). However, the preincubation of cells with fucoidan significantly reduced the integrated amplitude of glutamate-induced Ca^2+^ responses in cultured cortical neurons at both 0.5 and 1.5 mg/mL (Figure 1(A,E)). There was no significant difference between two different concentrations of fucoidan for attenuating glutamate-induced Ca^2+^ responses (Figure 1(A)).

Quisqualate (20 µM), a nonspecific agonist activating AMPA receptor (AMPAR) and ACPD receptor (Katz and Levitan 1993; Conn and Pin 1997), obviously increased the [Ca^2+^]*_i_* in cortical neurons, but the quisqualate-induced Ca^2+^ responses did not change significantly in cortical neurons treated with the experimental concentrations of fucoidan (Figure 1(B,E)). We also observed that fucoidan (0.5 mg/mL) has no significant effect on the AMPA-induced Ca^2+^ responses in cortical neurons (Figure 1(C,E)). However, the Ca^2+^ responses induced by another type of ionotropic receptor, NMDAR, were almost completely abolished by fucoidan (0.5 mg/mL) in the presence of extracellular 0.4 mM Mg^2+^ and 2 mM Ca^2+^ (Figure 1(D,E)).

**Figure 2.**
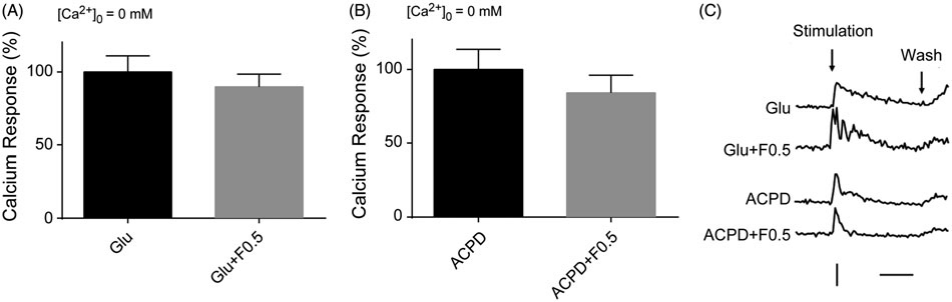
Effects of fucoidan on the Ca^2+^ responses induced by glutamate receptor agonists in the absence of extracellular Ca^2+^ in hippocampal neurons. Quantification of the areas of Ca^2+^ response curves was taken from all records in each test. The ordinate is expressed in reference to the control value. The error bars represent the SEM. (A) Ca^2+^ responses in hippocampal neurons induced by glutamate (50 µM) in the absence of extracellular Ca^2+^. (B) Ca^2+^ responses in hippocampal neurons induced by ACPD (100 µM) in the absence of extracellular Ca^2+^. The [Ca^2+^]*_i_* rises are evident after the wash, as the Ca^2+^ containing standard solution was used for washing to ensure the absence of extracellular Ca^2+^ during the test period. (C) Representative time course curves of the Ca^2+^ responses induced by glutamate (50 µM) and ACPD (100 µM) with or without fucoidan. Glu: glutamate; F: fucoidan. Vertical bar, the ratio of *F*_340_/*F*_380_ to be 1. Horizontal bar, 1 min. *n* = 3.

To elucidate the source of Ca^2+^ responses remaining after exposure to fucoidan, we examined the effect of fucoidan on [Ca^2+^]*_i_* in cortical neurons in the absence of extracellular calcium ions. Results showed that when calcium ions were removed from the extracellular medium by Ca^2+^ chelator, EGTA (1 mM), treatment of glutamate has no significant influence on [Ca^2+^]*_i_* in cortical neurons within the concentration range of 50–1000 µM (data not shown). Interestingly, glutamate (50 µM) could induce the Ca^2+^ responses in hippocampal neurons in the absence of extracellular Ca^2+^ (Figure 2(A,C)). Application of ACPD (100 µM), which activates both group I and II mGluRs (Irving et al. 1990; Conn and Pin 1997), did not induce Ca^2+^ transients either in the presence or absence of calcium in the cortical neurons (data not shown), but it did in the hippocampal neurons (Figure 2(B,C)). This was consistent with that of glutamate administration in the absence of extracellular Ca^2+^. These findings showed that glutamate or ACPD can induce Ca^2+^ releases from intracellular stores via activation of mGluRs which are expressed on the cell membrane of hippocampal. The increasing responses of [Ca^2+^]*_i_* induced by glutamate or ACPD in the absence of extracellular Ca^2+^ were insensitive to fucoidan treatment (0.5 mg/mL) (Figure 2(A–C)).

### Effects of fucoidan on the Ca^2+^ responses induced by Bay K8644 in neurons

**Figure 3.**
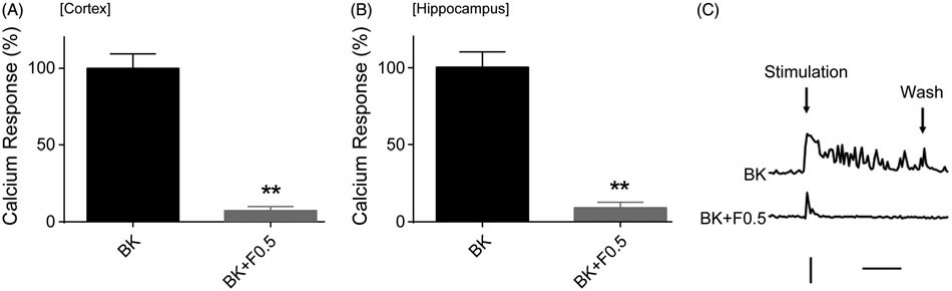
Effects of fucoidan on the Ca^2+^ responses induced by Bay K8644 in neurons and HeLa cells. (A) Quantification of the areas of Ca^2+^ response curves corresponding to the application in cortical neurons. (B) Quantification of the areas of Ca^2+^ response curves corresponding to the application in hippocampal neurons. (C) Quantification of the area of Ca^2+^ response curve examined in HeLa cells for comparison. The ordinate is expressed in reference to the control value. The error bars represent the SEM. **p < 0.01 vs. the untreated group. (C) Representative time course curves of the Ca^2+^ responses induced by Bay K8644 with or without fucoidan. For the control, Bay K8644 (20 µM) was applied (for 3 min) after recording the baseline for 3 min. For the test, fucoidan was added to the aCSF during the same baseline period (3 min), and then without removal of fucoidan, the agonists were further added to aCSF. BK: Bay K8644; F: fucoidan. Vertical bar, ratio of *F*_340_/*F*_380_ to be 1. Horizontal bar, 1 min. *n* = 3.

Bay K8644 is an opener of all l-type Ca^2+^ channels. In the presence of this agonist, Ca^2+^ channels tend to open for longer periods causing an increase in [Ca^2+^]*_i_*. Administration of Bay K8644 (20 µM) induced rapid Ca^2+^ responses in cortical and hippocampal neurons (Figure 3(A,B)). The Ca^2+^ responses were inhibited greatly by fucoidan at a concentration of 0.5 mg/mL (Figure 3(A,B)). Moreover, HeLa cells were challenged to Bay K8644 (20 µM), but Bay K8644 failed to induce a marked Ca^2+^ response (data not shown).

### Effects of fucoidan on the Ca^2+^ responses induced by adrenaline in hippocampal neurons

**Figure 4.**
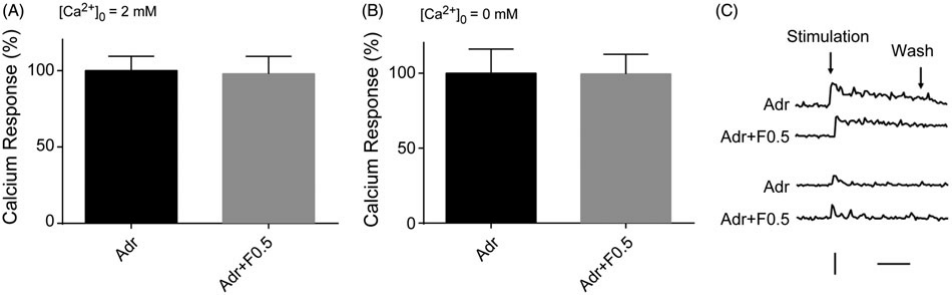
Effects of fucoidan on the Ca^2+^ responses induced by adrenaline in hippocampal neurons. Quantification of the areas of Ca^2+^ response curves corresponding to the application of adrenaline (100 µM) in hippocampal neurons in the presence (A) or absence (B) of extracellular calcium ions. The ordinates are expressed in reference to the control value. The error bars represent the SEM. Representative time course curves of the Ca^2+^ responses induced by adrenaline (100 µM) with or without fucoidan (C). Adr: adrenaline; F: fucoidan. Vertical bar: 0.5 (as ratio of *F*_340_/*F*_380_); horizontal bar: 1 min. *n* = 3.

Above results showed that fucoidan has no inhibitory effects on the mGluRs-mediated Ca^2+^ responses in neurons. This is inconsistent to our previous study which indicated that fucoidan mainly worked on G-protein-coupled receptors in HeLa cells. We wanted to confirm more about the effects of fucoidan on different G-protein-coupled receptors, for example, adrenergic receptors, expressed in hippocampal neurons. Adrenergic receptors are members of the G-protein-coupled receptor superfamily, and their activation causes an increase in inositol 1,4,5-trisphosphate (IP_3_) and [Ca^2+^]*_i_*. In hippocampal neurons, adrenaline (100 µM) treatment could apparently induce the Ca^2+^ responses in the both presence and absence of extracellular Ca^2+^ (Figure 4(A–C)); meanwhile, preincubation with fucoidan at 0.5 mg/mL failed to suppress the adrenaline-induced Ca^2+^ responses either in the presence of extracellular Ca^2+^ or in the absence of extracellular Ca^2+^ (Figure 4(A–C)).

### Effects of fucoidan on mRNA levels of iGluRs and mGluRs in neurons

**Figure 5.**
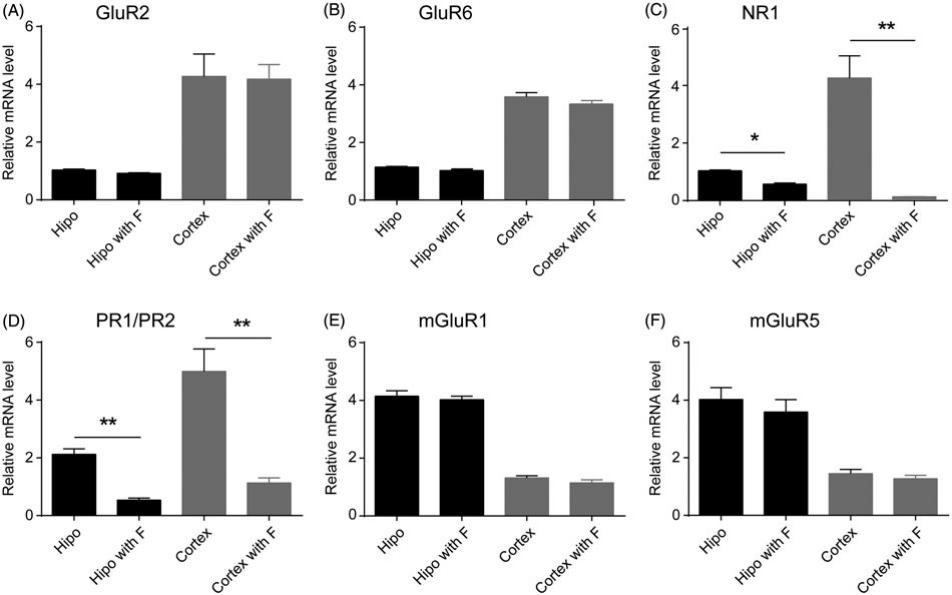
Effects of fucoidan on expressions of iGluRs and mGluRs in neurons. (A–C) Bar graph showed the ratio of iGluRs mRNA to β-actin mRNA in the untreated cells, or the cells treated with fucoidan (0.5 mg/mL) as indicated. (D) Bar graphs showed the ratio of PR1/PR2 mRNA to β-actin mRNA in the untreated cells or the cells treated with fucoidan (0.5 mg/mL). (E, F) Bar graphs showed the ratio of mGluR1 and mGluR5 mRNA to β-actin mRNA, respectively, in the untreated cells, or the cells treated with fucoidan (0.5 mg/mL). *p < 0.05 or **p < 0.01 vs. the untreated group. Hipo: hippocampal neuron; cortex: cortical neuron; F: fucoidan. *N* = 3.

Based on sequence homology, the pharmacology of agonist binding and the second messenger system, the mGluRs are divided into three subgroups (I, II and III) (Conn and Pin 1997). The group I mGluRs, i.e., mGluR1 and mGluR5 are thought to play a pivotal role in Ca^2+^ homeostasis (Sourial-Bassillious et al. 2009; Sevastyanova and Kammermeier 2014). Similarly, the iGluRs are classified into three groups based on structural similarities: NMDARs, AMPARs and kainate receptors (KARs). For the NMDARs, NMDAR subunit NR1 plays a critical role in modulating calcium conductivity of the channel (Traynelis et al. 2010). Moreover, AMPA subunit GluR2 is necessary for determinating the calcium permeability of the AMPARs (Lau and Tymianski 2010). KARs subunit GluR6 stimulation enhanced greatly Ca^2+^ responses (Ouardouz et al. 2009). PR1/PR2 is a primer pair for l-type channels (Perez-Reyes et al. 1990), sensitive to Bay K8644. In search for evidence that fucoidan suppresses the agonist-induced Ca^2+^ response by inhibiting specific receptors and not by quenching agonist molecules in the medium, we measured an intracellular response other than Ca^2+^ response. As we assumed that fucoidan binding to membrane receptors would induce some change in receptor expression and internalization (recycling), we measured the mRNA expressions of iGluRs (i.e., GluR2, GluR6 and NR1), PR1/PR2 and mGluRs (i.e., mGluR1 and mGluR5) using RT-PCR. Results showed that after 3 h of exposure to fucoidan (0.5 mg/mL), no apparent change of the mRNA expressions of GluR2 and GluR6 was observed in cortical and hippocampal neurons (Figure 5(A,B)). However, fucoidan significantly decreased the mRNA expressions of NR1 and PR1/PR2 in the cultured neurons (Figure 5(C,D)). Furthermore, the mRNA expressions of mGluR1 and mGluR5 showed a slight but not significant decrease following fucoidan treatment for 3 h (Figure 5(E,F)). These data suggested that fucoidan suppressed the agonist-induced Ca^2+^ responses in neurons by regulating the NMDARs and l-type Ca^2+^ channels.

## Discussion

In the current study, our data demonstrated that fucoidan attenuated the intracellular Ca^2+^ responses of neurons by modulating NMDARs and Bay K8644-sensitive Ca^2+^ channels, but not AMPA/KARs or metabotropic glutamate receptors (see Table 2). Glutamate induces the Ca^2+^ influx through the cell membrane by activating iGluRs, and also induces the Ca^2+^ release from intracellular stores by activating mGluRs. The iGluRs agonists NMDA, AMPA and glutamate could effectively increase the [Ca^2+^]*_i_* in cortical neurons with extracellular Ca^2+^. Under this condition, fucoidan inhibited glutamate-induced Ca^2+^ responses by 50%, NMDA-induced ones by 100%, and AMPA-induced ones by 0%. In the absence of extracellular Ca^2+^, hippocampal neurons, bearing mGluRs, showed rises of the [Ca^2+^]*_i_* when glutamate or ACPD (an mGluRs agonist) was applied, which fucoidan failed to inhibit. This indicates that the bulk of the glutamate-induced Ca^2+^ transients in cortical neurons was mediated by simultaneous activation of the NMDARs and Ca^2+^ permeable AMPA/KARs, and also that about 50% reduction of Ca^2+^ responses by fucoidan was ascribed to the inhibition of iGluRs of NMDARs but not of AMPARs in primary cultured neurons. The mGluRs are present on the cell membrane of hippocampal neurons, and they are insensitive to fucoidan. At the same time, Bay K8644 induced the Ca^2+^ responses in the presence of extracellular Ca^2+^, and the Ca^2+^ responses are suppressed by fucoidan by more than 90%. Similarly to NMDARs, l-type Ca^2+^ channels themselves are sensitive to fucoidan.

**Table 2.** Spectrum of effects of fucoidan.

	Neuron						
Agonist	Bay K8644	NMDA	Glutamate	ACPD	Quisqualate	Adrenaline	AMPA
GPCR	/	/	–	–	–	–	/
Ion channel	**+**	**+**	**+**	/	–	/	–

GPCR: G-protein-coupled receptor; **+**: inhibitory effect; –: no effect; /: no match.

Although both NMDA and AMPARs are belong to subfamilies of iGluRs based on their pharmacological and structural similarities, they showed different sensitivities to fucoidan. Literature of cloning indicates that the iGluRs have a large extracellular domain (the amino-terminal and agonist-binding domains), a transmembrane domain, and an intracellular C-terminal domain (Moriyoshi et al. 1991; Nakagawa et al. 2005). The extracellular domain of NMDAR contains about 60 lysine and arginine (Moriyoshi et al. 1991) where the binding sites for the sulphated residues of fucoidan may exist. Their amino acid-amino acid interval distributes in characteristic manners. Although the specific binding sites for fucoidan have not yet been determined, the difference in such interval between the positively charged amino acids would be a decisive factor for all-or-non effect of fucoidan on receptors. A known difference between the NMDAR and the AMPA/kinate receptor also lies in their positive charges in the transmembrane segment II (TMII) that dominates the functional characteristics including the Ca^2+^ permeability of receptors. In fact, an asparagine residue occupies the place in NMDAR which is equivalent to the glutamate-arginine editing site in AMPA/kinate receptors (Nakanishi 1992).

So far, the molecular weight of fucoidan is known not as a single value but rather a broad distribution around an average. In the present study, we used commercially available fucoidan purified from *Fucus vesiculosus* (Sigma), which is a heterogeneous mixture of more than 15 different fucans with varied proportions of individual monosaccharide (Nishino et al. 1994; Irhimeh et al. 2005). The molecular weight is about 20 kDa, the length roughly 40 nm, and the number of negative charges was estimated to be 80. Many studies have attempted to screen fucoidanases from marine polysaccharide-containing plants or bacteria which are the useful tools for cleaving off oligosaccharides from the end of the polysaccharide chain or cleaving somewhere in the middle of the polysaccharide to yield lower molecules and to decide the molecular weights (see the review, Holtkamp et al. 2009; Kusaykin et al. 2016). The functional properties of fucoidan have been suggested to depend on its structure (Berteau and Mulloy 2003; Ferreira et al. 2015). When the structure of fucoidan is determined clearly, its pharmacological potential will be evaluated according to the standard characteristics. One point to be noted at the moment is that each single bond that links fucans can be axially rotated to fit the whole chain of fucoidan molecule to a certain steric arrangement of positively charged amino acid residues of the receptor proteins. This versatility of fucoidan may explain the interaction of fucoidan with diverse membrane receptors.

## Conclusions

The present study shows that fucoidan suppresses the intracellular Ca^2+^ responses of neurons by selectively inhibiting NMDARs in cortical neurons and l-type Ca^2+^ channels in hippocampal neurons. However, further studies are needed to determine how fucoidan regulates the specific receptors. Since fucoidan alone influences the mRNA expression in the neurons, fucoidan is proven to bind these cells probably at specific receptor sites. A wide spectrum of fucoidan binding to cell membrane may be useful for designing a general purpose drug in future.
